# Coughing

**Published:** 1880-05

**Authors:** 


### COUGHING

A gentleman called upon us recently, who actua.ly escaped from the fangs of consumption some years ago ; and we are induced to present the circumstances.

" You speak of coughing continually. Let me suggest to you the query, whether this is not unnecessary and injurious ? I have long been satisfied, from experience and observation, that much of the coughing, which precedes and attends consumption, is voluntary. Several years ago, I boarded with a man who was in the incipient stages of consumption. I slept in a chambei over his bed-room, and was obliged to hear him cough continually and distressingly. I endured the annoyance, night after night, till it led me to reflect whether something could not be done to stop it. I watched the sound which the man made, and observed that he evidently made a voluntary effort to cough. After this I made several experiments on myself, and found that I could prevent myself from coughing, sneezing, gaping, &c., in case of the strongest propensity to these acts, by a strenuous effort of the will. Then I reflected that coughing must be very irritating and injurious to the delicate organs that are concerned in it, especially when they are in a diseased state. What can be worse for ulcered bronchia, or lungs, than the violent wrenching of a cough ? It must be worse than speaking. A sore on any part of the body, if it is constantly kept open by violent usage, or made raw again by a contusion just when it is healing (and of course begins to itch) will grow worse, and end in death. Certainly, then, a sore on the lungs may be expected to terminate fatally, if it is constantly irritated, and never suffered to heal ; and this, it seems to me, is just what coughing does foi it. On the strength of such considerations as these, I made bold to ask the man if he could not stop coughing. He answered, no. I told him what I thought about it, as above. He agreed to make a trial ; and on doing so, he found to his surprise that he could suppress his cough almost entirely. The power of the will over it increased as he exercised it, and in a few days he was mostly rid of the disposition to cough. His health, at the same time, evidently improved, and when I last saw him, he was in strong hopes of getting out of death's hands."

